# Development of Inkjet Printable Formulations Based on Polyorganosilazane and Divinylbenzene

**DOI:** 10.3390/polym15234512

**Published:** 2023-11-23

**Authors:** Afnan Qazzazie-Hauser, Kirsten Honnef, Thomas Hanemann

**Affiliations:** 1 Laboratory for Materials Processing, University of Freiburg, 79110 Freiburg, Germany; kirsten.honnef@imtek.uni-freiburg.de; 2 Institute for Applied Materials, Karlsruhe Institute of Technology (KIT), 76344 Eggenstein-Leopoldshafen, Germany

**Keywords:** polyorganosilazane, divinylbenzene, inkjet printing, UV-LED photopolymerization, electrical conductivity, free carbon, FTIR, Raman, TGA

## Abstract

Within this work, ink formulations based on polyorganosilazane (OPSZ) and divinylbenzene (DVB) were developed to be processed by inkjet printing. The formulations were studied regarding their rheological, structural, and thermal properties. The rheological results show that the new formulations meet the requirements of the inkjet printer by showing both low viscosity (below 20 mPa∙s at printing temperature) and Newtonian flow behavior even at high shear rates. Additionally, the inks have surface tensions in the range of 21 to 26 mN/m^2^. First, printing experiments of single layers were successfully conducted and show that the developed formulations can be processed by inkjet printing. The inks were crosslinked by UV light and then pyrolyzed at 1100 °C resulting in a ceramic yield between 75 and 42%, depending on the ink formulation. The crosslinking behavior was studied via FTIR spectroscopy, and the results reveal that crosslinking occurs mainly via free-radical polymerization of the vinyl group. Furthermore, the results indicate that silicon carbonitride (SiCN) was formed after the pyrolysis. The results of the electrical properties of the amorphous ceramics differ in dependence on the amount of DVB in the formulation. A maximum electrical conductivity of 1.2 S/cm^−1^ was observed for a UV-cured sample with a high amount of DVB pyrolyzed at 1100 °C. The generation in electrical conductivity is given by the formation of free carbon derived most likely by DVB.

## 1. Introduction

Polymer-derived ceramics (PDCs) are an outstanding class of material due to their ease of processability since they are processed in the same way as polymers, unlike standard ceramic materials [1]. They also offer several desirable characteristics, including thermal and chemical stability [2], excellent adherence to multiple surfaces [3,4,5], and the tunability of material properties. These characteristics allow for their use in a wide range of applications in micro-electro-mechanical systems (MEMS), biomedical implants, harsh environment sensors, and fuel cells [6,7,8,9,10,11].

Additive manufacturing offers a sustainable and versatile way to produce complex 3D components. Many applications can already be found in areas such as medical devices [12,13], automotives [14,15], and aerospace [16,17]. 3D printing techniques such as vat photopolymerization, which includes digital light processing [18,19,20,21] and stereolithography [22,23], fused deposition modeling [24,25], direct ink writing [26,27], and inkjet printing [28], are already being applied on advanced ceramic materials such as preceramic polymers [29,30]. The technique being used in this work is inkjet printing. In comparison to the above-mentioned 3D printing methods, several materials can be deposited in one step by inkjet printing. This allows for the rapid production of a fully functional component. Furthermore, inkjet printing is a low-cost method, as only small quantities of material are needed and almost no waste is generated [31]. The inks can be developed in terms of the desired property. A wide range of liquid and low-viscosity materials are deposited layer-by-layer by a drop-on-demand approach (DOD), where the droplets are generated by piezoelectric actuator [32]. The inkjet printing process is limited by a viscosity between 1 mPa∙s and 25 mPa∙s at printing temperature and a surface tension in the range of 25–50 mN/m [31,33]. The surface tension must be sufficiently high to prevent undesirable dripping from the nozzle. However, it must be low enough to release the jetted droplet from the nozzle [31]. An accuracy of about 30 µm can be achieved due to the small nozzle diameter [33]. After the deposition, the material is cured through photochemical or thermal crosslinking [34] in dependence on the used material.

This work focuses on polysilazanes, which are liquid silicon-based preceramic polymers with an alternating silicon and nitrogen backbone. They are referred to as poly-organosilazanes if they consist of alkyl or vinyl as a side group [35]. In addition, they contain hydrogen as a side group [36]. Polyorganosilazanes can be formulated with initiators, catalysts, or fillers to form processable matrix composites that can be fabricated by casting [37,38], coating [4,39], or additive manufacturing [18,40,41,42,43]. Crosslinking is usually carried out thermally [44,45] or photochemically [46] after processing to reduce the loss of low-molecular-weight components and consequently leading to a high ceramic yield after pyrolysis at high temperature [47].

Divinylbenzene (DVB) can be used as crosslinking agent which also acts as a carbon supplier for preceramic polymers. A radical initiator can start a vinyl polymerization of DVB with itself or with the vinyl groups of polyorganosilazane [48]. The formation of carbon chains within the precursor lead to free-carbon clusters after the pyrolysis process, which generates the electrical conductivity in the final ceramic. By improving the conductivity, a wide range of applications can be served, especially in MEMS applications. Moreover, the addition of a crosslinking agent can enhance the ceramic yield because it reacts with the volatile species of the precursor. Furthermore, this leads to less shrinkage in the final product.

The aim of this work was the development of new ink formulations based on polyorganosilazane and DVB suitable for inkjet printing.

## 2. Materials and Methods

### 2.1. Materials

**Figure 1:**
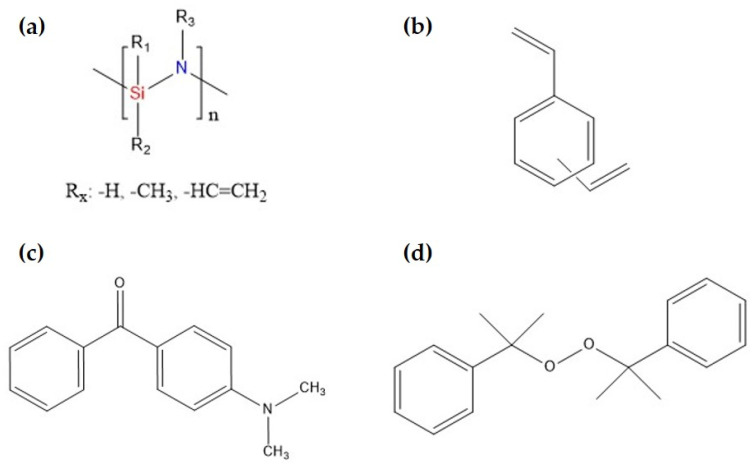
(**a**) Chemical structure of OPSZ with hydrogen, methyl, or vinyl bonds as side groups, (**b**) divinylbenzene, (**c**) the photo initiator 4-(dimethylamino)benzophenone, and (**d**) the thermal initiator dicumyl peroxide.

In this work, a commercially available liquid polyorganosilazane (OPSZ, Durazane 1800, Merck KGaA, Darmstadt, Germany) was used as preceramic polymer. Figure 1 presents the chemical structures of all components. The benzophenone derivate 4-(dimethyl-amino)benzophenone (DMABP, purity 98%, Merck KGaA, Darmstadt, Germany) and dicumyl peroxide (DCP, 98%, Alfa Aesar By Thermo Fisher Scientific, Waltham, MA, USA) were used as photo- and thermal initiator, respectively. The thermal initiator was used to ensure post-polymerization of the uncured groups of the thermoset after UV curing as it starts the crosslinking reaction at a temperature of 120 °C [49]. The surface additive Byk-3760 (Byk Additives & Instruments, Wesel, Germany) was used to improve the wetting behavior of ink. Divinylbenzene (DVB, purity 80% isomer mixture, Merck KGaA, Darmstadt, Germany) was used as crosslinking compound and carbon supplier. All materials were used as received.

### 2.2. Preparation of UV-Curable Inks and SiCN Ceramics

The samples were prepared by mixing the photo initiator DMABP (3 wt.%), the thermal initiator DCP (3 wt.%), and the surface additive Byk-3760 (0.5 wt.%) with divinylbenzene. The solid components were dissolved by using a high shear dispenser (T-10 basic Ultra-Turrax^®^, IKA, Staufen, Germany) for 120 s at 9400 rpm. After the dissolution of the photo- and thermal initiators, OPSZ was added and mixed by the high shear dispenser for another 60 s. Table 1 gives a complete sample description.

All samples were cured by UV irradiation using a LED light source (LED-Spot-100 lamp, Dr. Hönle UV Technology, Gräfelfing, Germany) with a wavelength of maximum emission at 385 nm for 300 s. The LED light source intensity I_0_ (569 mW/cm^2^) was measured by a UV-meter (HighEnd, Dr. Hönle UV Technology, Gilching, Germany).

The UV chamber was flooded by nitrogen gas to decrease the relative humidity (1.1% RH), which was measured by a Testo 608-H2 thermo hygrometer (Testo SE & Co., KGaA, Titisee-Neustadt, Germany), and to reduce the oxygen atmosphere around the sample.

For the electrical conductivity measurements, green bodies were produced using a silicone mold with a circular cavity. The formulations were filled into the mold by a micro-pipette with a volume of 100 µL for each layer and were then irradiated with UV light for 300 s. The green bodies consist of three layers, because this was the only way to ensure deepness through curing. The silicone mold was coated with PTFE to reduce the adhesion of the green bodies to the silicone and to facilitate the removal. After crosslinking, the circular green bodies were released from the mold with a diameter of 1.2 mm and a thickness of 1 mm. Pyrolysis was carried out at 1100 °C in an alumina tube furnace (Carbolite, Neuhausen, Germany) under a constant nitrogen flow. The samples were placed in an aluminum oxide crucible and were moved to the center of the tube furnace. The heating rate was 1 K/min up to 1100 °C followed by a holding time of 5 h. The cooling rate was 5 K/min.

The formulations were printed on a silicon wafer by using a PixDro LP50 inkjet printer (Süss Microtec, Garching bei München, Germany) conducted with the printhead DMC-11610 cartridge (Fujifilm Dimatix Inc., Santa Clara, CA, USA) with a drop volume of 10 pl. The printer was flooded with nitrogen to reduce the relative humidity during the printing process. The printed layer was UV irradiated for 300 s and then pyrolyzed at 1100 °C in nitrogen atmosphere.

### 2.3. Characterization

The crosslinking behavior and the chemical composition of all samples were investigated by Fourier transform infrared (FT-IR) spectroscopy. A Bio-Rad FTS 3000 Excalibur spectrometer (Varian, East Palo Alto, CA, USA) was used to record the spectra of all samples within a wavenumber range of 4000 and 500 cm^−1^ by averaging 32 scans at a resolution of 4 cm^−1^. A doctor blade was used to apply the samples on a shiny etched silicon wafer, which was then measured in an uncured state, after UV irradiation and after pyrolysis. The pyrolysis process was carried out in a nitrogen atmosphere in a tube furnace at maximal temperature of 1100 °C, as the wafer is sensitive to higher temperatures. The temperature was kept at 1100 °C for 5 h and the heating rate was controlled to 1 K/min.

Raman spectroscopy was performed by a confocal Raman microscope (WITec alpha300, WITec GmBH, Kroppach, Germany) using a 532 nm laser, which operated at 10 ± 1 mW as the excitation source. Three measurements were performed by each thin layer sample. Background subtraction was done by WITec project. The D- and G-bands, relating to the sp^2^- and sp^3^-hybridized carbons were fitted according to Lorentz. The ratio of the D-band and G-band intensities *I_D_*/*I_G_* was determined by the amplitude of each band. For amorphous carbon, the graphitic cluster size *L_a_* can be calculated as follows (1), according to Ferrari and Robertson [50], where *C*′(*λ*) is a wavelength-dependent pre-factor (for *λ* = 532 nm, *C*′(*λ*) = 0.0062 Å^−2^).

(1) $$\frac{I_{D}}{I_{G}}=C^{\prime }\left(\lambda \right)\times L_{a}^{2}$$

Thermogravimetric analysis (TGA) was carried out to evaluate the ceramic yield after the evaluation of the polymer to ceramic conversion. The TGA measurement of cured samples was conducted by using STA-409C (Netzsch Group GmbH &Co, Selb, Germany). A heating rate of 10 K/min was set to heat the cured samples up to 1100 °C in nitrogen atmosphere. The uncertainty of the measurement of the residual ceramic yield is around ±2%.

The viscosity measurements of the prepared inks were performed by a cone and plate rheometer (Bohlin CVO50, Malvern Panalytical, Malvern, United Kingdom), with a cone diameter of 60 mm, a cone inclination angle of 2° and agap size of 70 µm as function of the shear rate (2–500 s^−1^) at 25 °C. The viscosity of each ink was measured three times. The experimental uncertainty for the viscosity is ± 1 mPa∙s.

Surface tension and contact angle measurements of the inks were performed by Drop-Shape-Analysis (DSA 100, Krüss GmbH, Hamburg, Germany). Three measurements for each sample were performed for the surface tension and contact angle.

The electrical conductivity of the ceramics was measured at room temperature by using a collinear four probe and a Keysight B1500A Semiconductor Device Analyzer (Keysight Technologies, Inc., Santa Rosa, CA, USA). The spacing between the contact pins was 2.25 mm. The measurement was made by passing a current between 1 µA and 1 mA, depending on the sample, through the outer probes and measuring the respective voltage between the inner probes with a step between 0.01 and 0.1 mA in dependence of the sample. The ohmic resistance was calculated from the slope of the obtained data. The electrical resistivity ρ was calculated from the obtained resistance *R_mean_* (2):

(2) $$\rho =G\cdot R_{mean}$$

where *G* = 2πs, which is a geometric correction factor for semi-infinite volume. *G* is multiplied by an additional correction factor T_1_(t/s) dependent on the finite thickness t of the sample, which is tabulated in the literature [51]. The electrical conductivity σ is the inverse of ρ. The 4-point measurement was carried out on three specimens for each formulation. Each specimen was contacted and measured three times at different points.

## 3. Results and Discussion

### 3.1. Rheology and Wetting Behavior of the Ink

**Figure 2:**
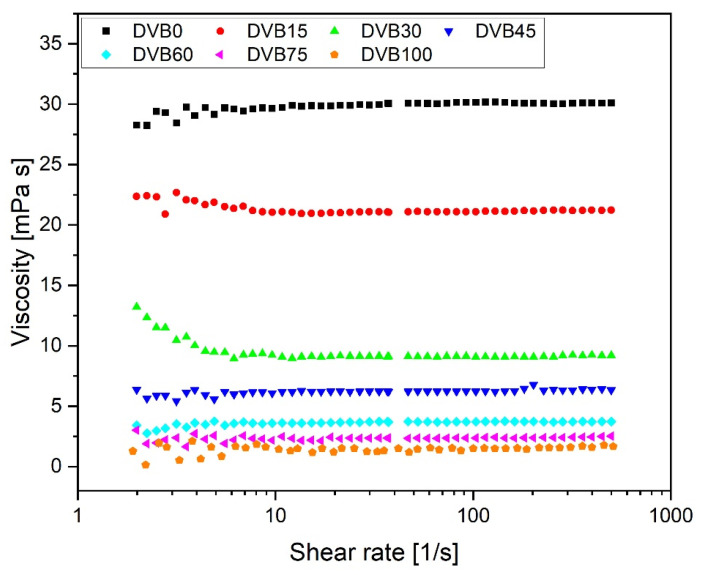
Viscosity of the inks measured in dependence on the shear rate at 25 °C.

The samples with various OPSZ/DVB ratios were characterized regarding their viscosity as function of shear rate at 25 °C. All samples exhibited the expected Newtonian behavior. With the increase in DVB content, the viscosity drops, given that pure DVB, which is referred to as DVB100, has a low viscosity around 1.6 mPa∙s, as Figure 2 shows. Table 2 lists the viscosity values for shear rates at 500 s^−1^. For inkjet printing, Newtonian behavior is beneficial for the inks, because the viscosity should remain constant at high shearing, which is the case during the printing process [33]. In addition, inkjet printing requires a viscosity below 20 mPa∙s under ambient conditions [33], which samples DVB0 and DVB15 do not meet. Therefore, these samples should be printed at a higher temperature, as the viscosity decreases with higher temperature [52]. All other samples can be printed at 25 °C, as they meet the requirements of the printer.

The surface tension is an important characteristic for inkjet printing, as it influences the droplet formation [16]. For DVB0, the surface tension was the lowest at 21 mN/m. By increasing the amount of DVB, the surface tensions of all the samples increase and are settled between 24 and 26.4 mN/m, which meets the requirements of inkjet printing which are in the range of 25–50 mN/m [16,17]. The reason for this increase is due to the surface tension of DVB which is 30.55 mN/m at 25 °C [53]. The results for the contact angle on the silicon wafer show a complete spreading and wetting of the formulations on the substrate. The wetting behavior was improved by the addition of the surface tension additive Byk-3760, which decreases the surface tension. 

### 3.2. Crosslinking Behavior and FTIR Analysis

**Figure 3:**
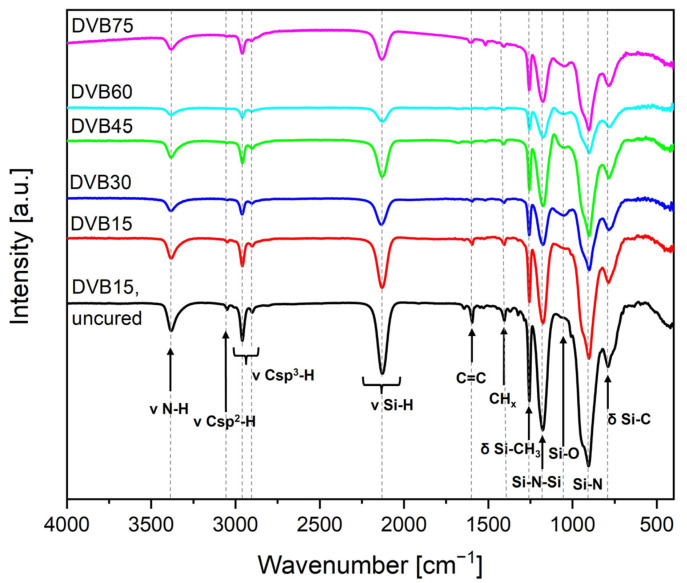
FTIR spectra of the uncured DVB15 sample and the cured samples with increasing amounts of DVB.

The FTIR analysis was performed to study the crosslinking behavior of the UV- cured samples and their resulting chemical structure. Figure 3 shows the FTIR spectra of the liquid and uncured DVB15 and of all crosslinked samples with an increasing amount of DVB. The assignment of the peaks was conducted in accordance with the literature [54]. As already described in our previous work [14], the stretching vibrations of the N-H and Si-H bonds (3382 and 2135 cm^−1^), respectively, and the stretching and the deformation vibration of the vinyl groups (3046 and 1595 cm^−1^) are responsible for the crosslinking process in OPSZ. The spectra of the UV-cured samples show that bands corresponding to the vinyl group decrease and, in some cases, disappear, which is attributable to the radical polymerization of the vinyl groups in OPSZ and DVB [46,48]. The radicals of the photo initiator activate the vinyl groups in DVB and OPSZ and initiate the polymerization, where DVB monomers can be integrated into the OPSZ crosslinking network. As Figure 3 shows, the bands of the stretching vibrations of the N-H and Si-H bonds at 3382 cm^−1^ and 2135 cm^−1^, respectively, remain after UV polymerization implying an incomplete crosslinking of the system. For this reason, the thermal initiator DCP was added to the formulations to post-cure the samples during the pyrolysis process in a nitrogen atmosphere. For this curing step, a dwell time of 30 min at 150 °C was set and afterwards, a slow heating rate of 1 °C/min was adjusted to enable transamination and dehydrocoupling reactions.

The crosslinking behavior was studied by taking a closer look at the reactive vinyl group. The relative degree of conversion DC (%) of the reactive vinyl group was calculated as described in our previous work [14]. As a reference, the sharp band of Si-CH_3_ at 1256 cm^−1^ was taken, since it is a characteristic band for OPSZ and is not involved in the crosslinking process. Table 3 summarizes the results of the degree of conversion and they are as high as 83%, indicating free-radical polymerization of vinyl groups during the crosslinking process induced by the LED source (λ_max_ = 385 nm). This result is evident in the spectra (Figure 3) since the band of the vinyl group at 1595 cm^−1^ decreases for all cured samples. The results for the degree of conversion of the Si-H and N-H bonds were neglected because their calculation from the spectra could not be performed with certainty for all samples.

**Figure 4:**
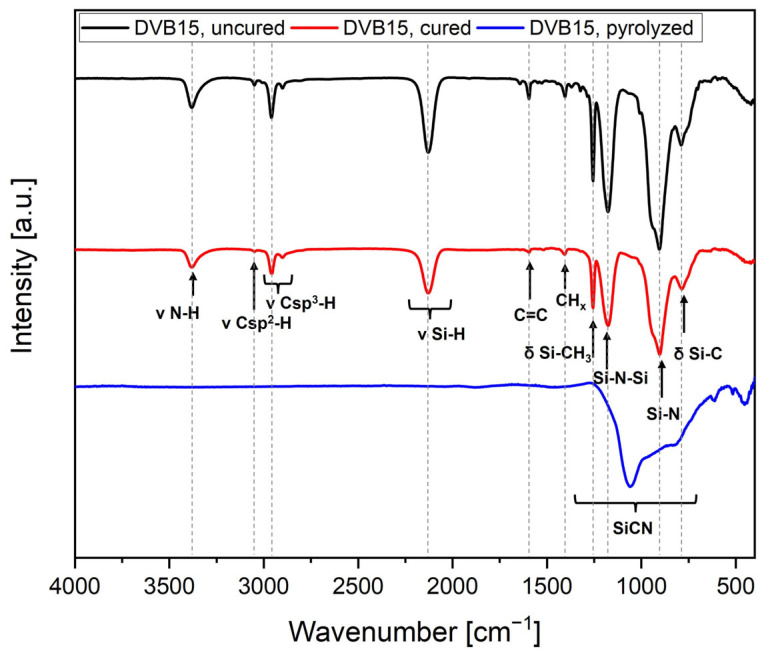
FTIR spectra of uncured, cured DVB15, and the corresponding amorphous ceramic SiCN pyrolyzed at 1100 °C (red curve).

Figure 4 shows the plotting of the FTIR spectra of the uncured, the cured, and the pyrolyzed samples of the formulation DVB15.

The bands of the organic bonds disappear after pyrolysis at 1100 °C due to a complete polymer to ceramic conversion. The ceramization process leads to an amorphous ceramic network at the given temperature. The broad band between 1250 and 600 cm^−1^ remained present corresponding to Si-O-Si, Si-N-Si, and Si-C bonds. The presence of oxygen in the amorphous ceramic is due to the sensitivity of OPSZ to moisture leading to hydrolysis and polycondensation reaction [55]. Within this work, it cannot be prevented because the samples were prepared in an ambient atmosphere.

### 3.3. Thermal Analysis and Ceramization Process

**Figure 5:**
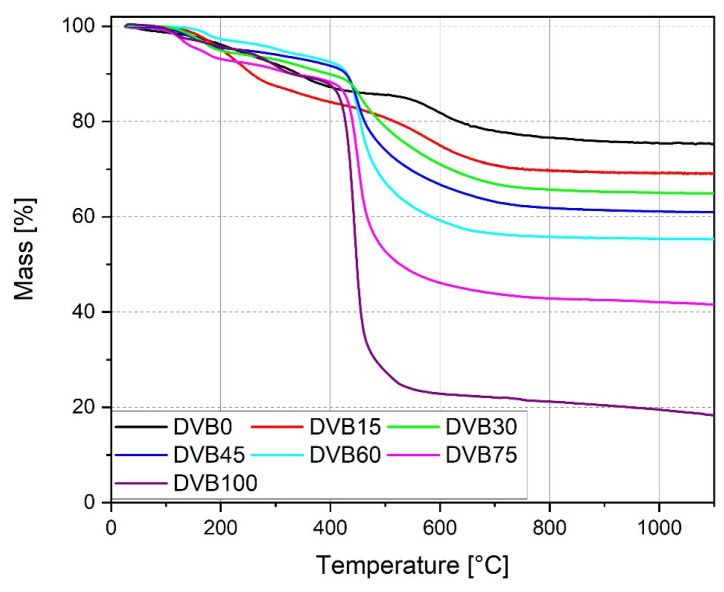
Thermograms of the samples cured by UV-LED source (λ_max_: 385 nm, 300 s, N_2_).

Thermogravimetric analysis was performed for all cured samples, including pure UV-cured OPSZ and DVB (DVB0 and DVB100), and DVB15–DVB75 to analyze the thermal decomposition of the samples during pyrolysis and to measure the ceramic yield. Figure 5 shows the thermograms. 

All cured samples show a similar two-step decomposition process. The first step occurs between 100 and 300 °C, which is caused by the evaporation of non-crosslinked volatile low-weight oligomers. The mass loss in the first step is observed, because of the crosslinking process, which was performed completely by UV irradiation. If the samples were cured thermally [48], the evaporation of the volatile low-weight oligomers will take place during the crosslinking process and will not be visible in the thermogram. That is the reason why the ceramic yield result of sample DVB60 is around 10% lower in comparison to the literature [48]. The second step occurs between 400 and 650 °C for the samples DVB30, DVB45, DVB60, and DVB75, whereas for samples DVB0 and DV15, it begins around 550 °C. The mass loss is caused by the pyrolysis of the organic skeleton, which was induced by the decomposition of the crosslinked OPSZ and DVB resulting in the organic–inorganic transformation of the thermoset leading to amorphous SiCN. The weight changes above 800 °C remained constant, which indicates that the transformation from a thermoset to a ceramic was complete. By increasing the amount of DVB, the second step starts earlier due to the higher number of volatile organic components and the residual mass decreases after the pyrolysis. Table 4 lists the residual mass for each sample as percentage of the total mass at 1100 °C. 

As expected, DVB75 exhibits the highest mass loss regarding the OPSZ/DVB inks, resulting in a ceramic yield of 42%. The reason for this result is due to the low amount of the preceramic polymer OPSZ in the formulation. The residual mass of DVB100 is the lowest mass, as it consists of pure cured DVB. Since the samples were measured in a nitrogen atmosphere, the residual mass is supposed to be carbon.

### 3.4. Electrical Conductivity of the Bulk Material

**Figure 6:**
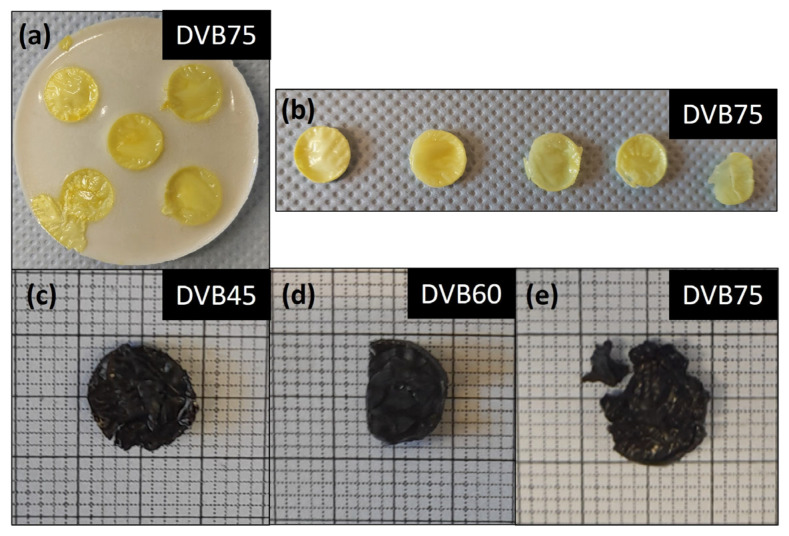
(**a**) UV-cured specimens of DVB75 in silicon mold, (**b**) bulk specimens after taking out of the mold, and (**c**–**e**) bulk specimens of samples DVB45–DVB75 after pyrolysis at 1100 °C.

Electrical conductivity measurements were performed at room temperature for the samples DVB45, DVB60, and DVB75, since it is an important material property for possible applicability in MEMS devices. The bulk specimens for the measurement were first UV cured (Figure 6a,b) and then pyrolyzed at 1100 °C (Figure 6c–e). 

After UV curing, the specimens were yellowish, and the surface was uneven and bubbling. Despite spraying the mold with PTFE, the green bodies adhered to the side walls and were therefore separated from the walls by a scalpel. The cured test specimens measured 1.2 cm in diameter and 1 mm in thickness. The pyrolyzed specimens are shiny and black in color, due to the formation of free carbon along the Si-C-N matrix. As Figure 6c–e shows, the ceramic samples partly cracked after pyrolysis despite using a controlled pyrolysis program. Therefore, spring-loaded pins were used to contact the specimens during the 4-point measurement. 

Table 5 presents the electrical conductivity results of the pyrolyzed samples with different amounts of DVB and OPSZ. 

It was found that the electrical conductivity increases with the amount of DVB and the results were between 0.1 and 1.2 S/cm. This outcome is comparable with the amorphous carbon-rich C/SiCN nanocomposites prepared by Adigun et al. [56]. As well known from the literature, pure OPSZ samples are insulators at pyrolyzing temperature below 1000 °C [1,15]. Therefore, it can be concluded that the increase in electrical conductivity is mainly due to the addition of DVB, as it acts as a carbon supplier. In addition, the UV polymerization process prevents volatile groups from being evaporated before curing, thereby contributing to chain growth, which in turn leads to higher conductivity. The crosslinked DVB in the OPSZ network forms free carbon after pyrolysis, which is responsible for the electrical conductivity in SiCN ceramics. A certain amount of free carbon supplied by DVB is required to form a percolation network within the amorphous ceramic to be electrically conductive in this temperature range. 

### 3.5. Raman Spectroscopy

**Figure 7:**
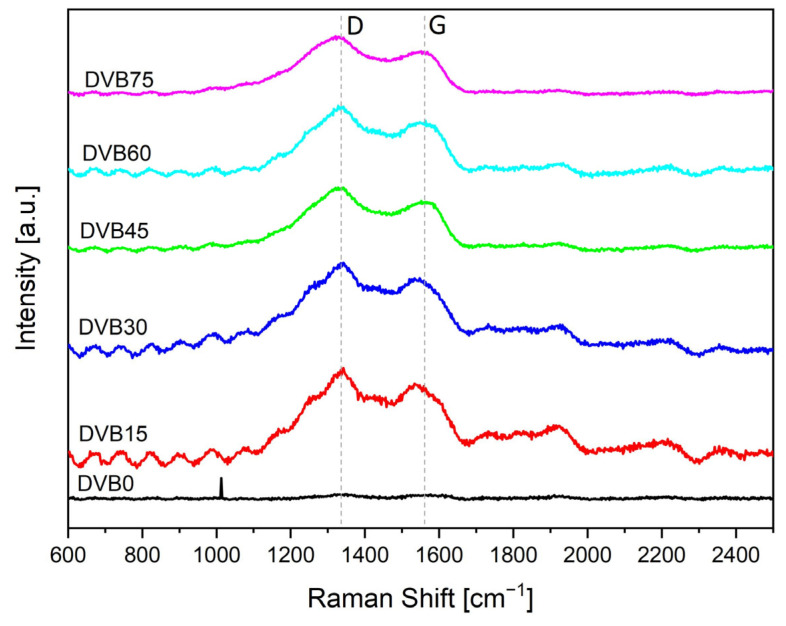
Raman spectra of SiCN thin film samples pyrolyzed at 1100 °C with different DVB content with the assignment of the D (disorder) and G (graphitic) bands.

Raman spectra (λ = 532 nm) were taken to confirm the presence of free carbon in the pyrolyzed samples (at 1100 °C). Figure 7 shows the plotting of the spectra of DVB15 to DVB75. 

Two sharp peaks can be seen in all spectra. The peaks are associated to the D (dis-order) and G (graphitic) bands of the free carbon phase. The band intensities and the peak center were determined by applying Lorentzian peak fitting on the background subtracted spectra due to the overlapping of the D and G band peaks. Table 6 lists the results. The D band peak shifts between 1320 and 1338 cm^−1^ and the G band occurs between 1535 and 1563 cm^−1^, depending on the structural order. Both bands are associated to the sp^2^ sites [19]. The G band occurs in all sp^2^ sites, both in aromatic and olefinic molecules, as it is referred to the in-plane bond stretching mode of pairs of carbon. Whereas the D band is only active in the presence of disorder-induced vibrations and its presence is strictly connected to the presence of sixfold aromatic rings [19], which means that the vinyl groups of DVB were incorporated by radical polymerization into the OPSZ crosslinking network forming free carbon clusters containing aromatic rings after the pyrolysis process. According to the three-stage model of Ferrari and Robertson [19], the carbon present is a mixture between nanocrystalline and amorphous carbon, because the G band is below 1600 cm^−1^. 

Table 6 lists the I_D_/I_G_ ratio and the size of free carbon cluster L_a_ calculated using Equation (1). It is found that the I_D_/I_G_ ratio increases with the addition of DVB to the system with the highest ratio calculated for DVB45, correspondingly also the size of free carbon clusters. The increase signifies a higher degree of ordering of the aromatic layer in the system, which also explains the increase in electrical conductivity. 

The free carbon cluster size L_a_ increases with the addition of DVB but does not increase linearly with higher content. It is found that above 30% of DVB content, the clusters size remains constant at around 1.7 nm.

### 3.6. Inkjet Printing

**Figure 8:**
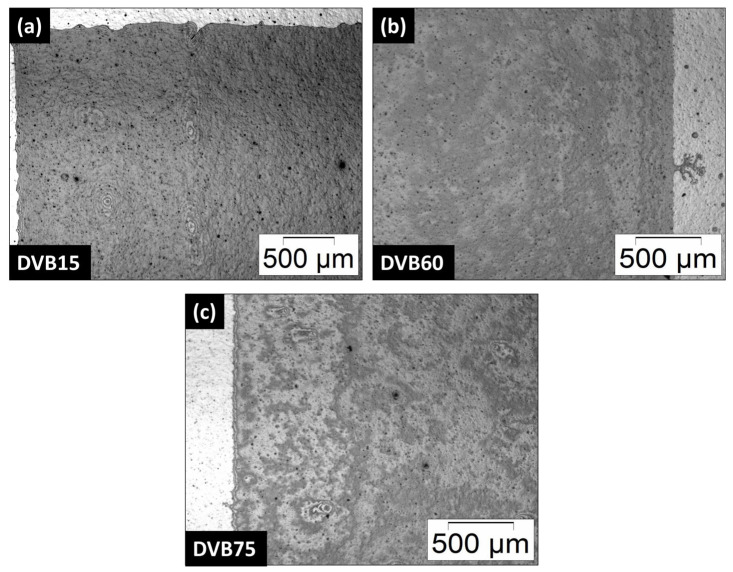
(**a**–**c**) Single thin layer of the inks DVB15 (40 °C), DVB60, and DVB75 printed on a silicon wafer by inkjet printing with a resolution of 450 × 450 dpi.

First, printing experiments of the developed formulations were conducted by a PixDro LP50 inkjet printer. All formulated inks were printable. Figure 8a–c shows the printed single layer of the inks DVB15, DVB60, and DVB75 on a silicon wafer with a resolution of 450 × 450 dpi. 

The temperature of the print head was 27 °C for the inks DVB60 and DVB75 and 40 °C for DVB15 due to higher viscosity. The relative humidity was 24% during the printing process. A higher amount of DVB reduced the viscosity of the inks, therefore, it was possible to print the layers at room temperature. Homogeneous closed single layers were successfully printed. There were little imperfections at the edges of the layers which were due to the non-ideal lab conditions, especially working in an ambient atmosphere. Although the inkjet printer was flooded with nitrogen, it was not fully inert and this can affect printing quality as OPSZ is sensitive to humidity. The humidity can affect the chemical structure of OPSZ due to hydrolysis and polycondensation reactions by forming silanol groups out of the Si-NH groups with water [55]. Figure 8a shows there were some holes in the layer of DVB15, which were due to failed nozzles. The big black points in the layers could have been caused by dust particles or impurities that may have been on the wafer and which cannot be prevented entirely.

The printed layers were dielectric after pyrolysis. The reason for that is that a thin single layer is insufficient to induce a percolation threshold of the free carbon and generate electrical conductivity. 

## 4. Conclusions

In summary, we have successfully developed new ink formulations based on polyorganosilazane (OPSZ) and divinylbenzene (DVB) for inkjet printing with a simple process route. The developed inks meet the requirements of the inkjet printer as they show Newtonian flow behavior, low viscosities, and low surface energies. In addition, the inks exhibit good wetting behavior and were characterized regarding their structural and thermal properties. All inks can be cured photochemically and then pyrolyzed at 1100 °C resulting in a ceramic yield between 75 and 42% in dependence on the ink formulation. The crosslinking process is mainly induced by free-radical polymerization of the vinyl groups as it was analyzed by FTIR spectroscopy. Due to the photo initiator 4-(dimethyl-amino)benzophenone, the formulations were cured by an LED light source (λ_max_ = 385 nm) in 300 s at room temperature under nitrogen flow. After the pyrolysis at 1100 °C, the FTIR spectrum indicate the formation of amorphous silicon carbonitride (SiCN). The electrical conductivity of bulk SiCN ceramics pyrolyzed at 1100 °C increases with respect to the DVB content and ranges between 0.1 and 1.2 S/cm. The introduction of DVB enhanced the electrical conductivity due to the formation of free carbon, as demonstrated by Raman spectroscopy. The characteristic D and G peaks of the free carbon phase were only visible in the samples including DVB. Nevertheless, only samples with a high amount of DVB (above 45%) were electrically conductive. 

Finally, the first inkjet printing experiments were successfully conducted. It was possible to print homogeneous single layers of the developed inks at room temperature and in a reduced ambient atmosphere. The printed layers were electrically non-conductive because of their low thickness. The generation of electrical conductivity with these formulations is, however, of great importance, as, for example, printed circuit boards can be processed in the future without the addition of conductive fillers or ceramics, which usually tend to clog the nozzles of the inkjet printhead. In general, preceramic polymers offer tremendous potential due to their easy processability and tailor-made properties. Their processability as polymers and their conversion into a ceramic after pyrolysis without the addition of a filler turn them into a sought-after class of materials for inkjet printing used in microsystems technology. 

It is necessary to conduct further experiments to explain the differences between bulk samples and thin layers in terms of electrical conductivity. Furthermore, samples with different resolutions will be printed and pyrolyzed to determine the minimum thickness at which electrical conductivity can be achieved.

## Figures and Tables

**Table 1:** Sample description. The sample names are referred to as DVB15, DVB30, and so on depending on the quantity of DVB.

Sample	OPSZ (wt.%)	DVB (wt.%)	Photo Initiator (wt.%)	Thermal Initiator (wt.%)
DVB0	100	0	3	3
DVB15	85	15	3	3
DVB30	70	30	3	3
DVB45	55	45	3	3
DVB60	40	60	3	3
DVB75	25	75	3	3

**Table 2:** Results of the surface tension, wetting, and rheological behavior at the highest shear rate of all samples.

Sample	Surface Tension (mN/m)	Contact Angle on Silicon Wafer (°)	Viscosity at 500 s^−1^ (mPa∙s)
DVB0	21	5	30
DVB15	24.7	5	21
DVB30	24.2	3	9.5
DVB45	24.9	Spreading	7
DVB60	24.6	3	3.7
DVB75	26.4	Spreading	2.5
DVB100	30.55	-	1.6

**Table 3:** The degree of conversion of the reactive vinyl group calculated from the FTIR spectra of all samples.

Sample	Degree of Conversion (Vinyl Group at 1595 cm^−1^) (%)
DVB0	-
DVB15	82 ± 3
DVB30	66 ± 7
DVB45	75 ± 7
DVB60	81 ± 3
DVB75	83 ± 9

**Table 4:** The residual masses of all samples were collected by TGA at 1100 °C.

Sample	Residual Mass at 1100 °C (%)
DVB0	75
DVB15	69
DVB30	65
DVB45	61
DVB60	55
DVB75	42
DVB100	17

**Table 5:** Electrical conductivities of DVB45, DVB60, and DVB75 pyrolyzed at 1100 °C.

Sample	Electrical Conductivity (S/cm)
DVB45	1.1 × 10^−1^
DVB60	2.3 × 10^−1^
DVB75	1.2

**Table 6:** Raman results fitted by Lorentz of the SiCN ceramics with different amounts of DVB. The standard deviation of the ratio is 0.1.

	D-Band (cm^−1^)	G-Band (cm^−1^)	I_D_/I_G_ -	L_a_ (nm)
DVB0	1338	1563	1.0	1.2
DVB15	1329	1541	1.3	1.5
DVB30	1320	1539	1.9	1.7
DVB45	1330	1546	2.0	1.8
DVB60	1327	1535	1.5	1.6
DVB75	1322	1547	1.8	1.7

## Data Availability

Data are contained within the article.
